# Cerebral Tuberculoma in a Patient With Pre-extensively Drug-Resistant Tuberculosis and AIDS: Diagnostic and Management Challenges

**DOI:** 10.7759/cureus.89546

**Published:** 2025-08-07

**Authors:** Sahak Mkrtchyan

**Affiliations:** 1 Department of Tuberculosis, Yerevan State Medical University After Mkhitar Heratsi, Yerevan, ARM

**Keywords:** central nervous system tuberculosis, cerebral tuberculoma, drug-resistant tuberculosis, extrapulmonary tuberculosis (eptb), hiv aids, hiv coinfection, immune reconstitution inflammatory syndrome (iris), immunocompromised patient, pre-extensively drug-resistant tuberculosis, tuberculosis

## Abstract

Extrapulmonary tuberculosis (TB), particularly when it involves the central nervous system (CNS), remains a significant clinical challenge. Cerebral tuberculoma, though rare, can present with complex symptoms that overlap with other neurological conditions, making timely diagnosis difficult. The condition demands a multidisciplinary approach for accurate diagnosis and effective management, especially in patients with multiple comorbidities. This report describes a complex case of pre-extensively drug-resistant TB affecting both the lungs and CNS in a 54-year-old immunocompromised male with AIDS, chronic hepatitis B and C, COVID-19, and reactivated varicella. The patient presented with systemic symptoms and new-onset neurological deficits, including seizures and right-sided paresis. Brain MRI revealed a cerebral tuberculoma with surrounding edema. Sputum testing confirmed *Mycobacterium tuberculosis* resistant to multiple first- and second-line agents. Treatment included a five-drug anti-tuberculosis regimen followed by initiation of antiretroviral therapy. The clinical course was complicated by immune reconstitution inflammatory syndrome, hepatic decompensation, and varicella reactivation. Despite profound immunosuppression and multisystem involvement, the patient achieved a favorable outcome through aggressive, multidisciplinary management. This case underscores the diagnostic complexity of CNS TB in patients with advanced HIV infection and multiple viral coinfections and highlights the critical role of early neuroimaging and coordinated care.

## Introduction

Tuberculosis (TB) is the leading cause of death from a single infectious agent worldwide [1] and continues to pose a significant global public health challenge affecting all populations, with a particularly high burden in low- and middle-income countries [2] and among immunocompromised individuals [3]. The intersection of TB with HIV and emerging pathogens such as SARS-CoV-2 presents substantial diagnostic and therapeutic challenges [4]. According to the World Health Organization’s 2020 report, TB remains the leading cause of death among people living with HIV [5], with central nervous system (CNS) involvement significantly increasing morbidity and mortality.

CNS tuberculosis accounts for approximately 1% of all TB cases and represents its most devastating manifestation [6]. One study suggested that the estimated mortality of CNS tuberculosis in hospitalized patients is approximately 42%, primarily based on meningitis-related deaths [7]. Cerebral tuberculoma, one of its forms, is particularly challenging to diagnose due to its nonspecific neurological symptoms and its radiological resemblance to neoplasms [8] or pyogenic abscesses. In patients with advanced HIV, the clinical presentation may be further complicated by opportunistic infections, drug interactions, and immune reconstitution inflammatory syndrome (IRIS) following the initiation of antiretroviral therapy (ART) [9].

This report describes a rare and life-threatening case of pre-extensively drug-resistant (pre-XDR) TB, defined as TB that meets the criteria for multidrug-resistant and rifampicin-resistant TB and is additionally resistant to any fluoroquinolone, with pulmonary and cerebral involvement in a patient with AIDS from the Caucasus region, where TB remains a persistent public health concern. The case was further complicated by chronic hepatitis B and C, COVID-19, and varicella, reflecting the full complexity of managing TB in immunosuppressed hosts. Special emphasis is placed on the cerebral tuberculoma, which was the most striking extrapulmonary feature and required careful neurological assessment and follow-up.

## Case presentation

A 54-year-old male construction worker was admitted to a specialized center for drug-resistant TB with complaints of persistent cough, exertional dyspnea, fatigue, night sweats, dizziness, right-sided weakness, and prolonged fever.

The patient’s symptoms began approximately four months prior with a persistent cough, malaise, and intermittent fever reaching 39°C. He initially self-medicated with antipyretics and delayed seeking medical attention. Over time, his condition deteriorated, and he later developed seizures and right-sided weakness.

**Figure 1:**
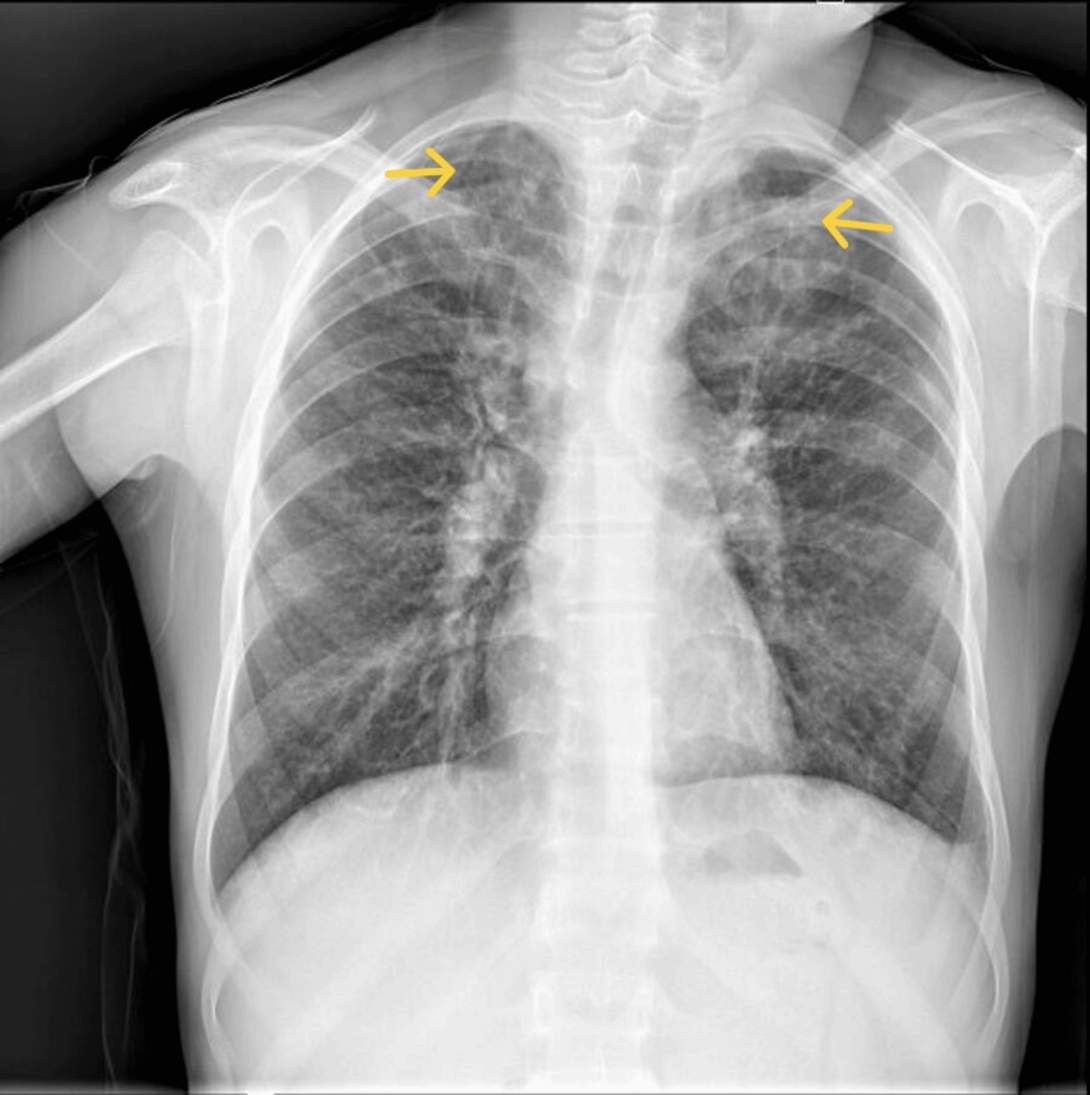
Frontal chest radiograph showing bilateral heterogeneous infiltrates and multiple cavitary lesions, most pronounced in the upper lobes. Arrows indicate the cavitations.

Chest radiography revealed heterogeneous infiltrates and cavitary lesions bilaterally, most prominently in the upper lobes (Figure 1). Sputum microscopy was positive for acid-fast bacilli (AFB, 2+), and GeneXpert testing confirmed the presence of *Mycobacterium* *tuberculosis* (MTB) DNA with rifampin resistance.

On examination, the patient had a body mass index of 21.1 kg/m^2^ (weight: 61 kg; height: 170 cm). His medical history included chickenpox one year prior, a 30-year history of smoking, and no known drug allergies. He had never been evaluated for HIV care. Physical examination revealed pale, diaphoretic skin, a coated tongue, and bilateral crackles in the upper lung fields. Oxygen saturation measured by pulse oximetry ranged from 92% to 95% on room air. Additional findings included hepatomegaly and right-sided hemiparesis.

Initial diagnostic testing was positive for SARS-CoV-2 by polymerase chain reaction (PCR). Sputum cultures on Löwenstein-Jensen and Mycobacteria Growth Indicator Tube media confirmed the presence of MTB. Drug susceptibility testing demonstrated resistance to isoniazid, rifampin, ethambutol, and fluoroquinolones, fulfilling the criteria for pre-XDR TB.

Additional laboratory evaluation confirmed HIV infection, with a viral load of 9 million copies/mL and a CD4+ T-cell count of 106 cells/µL. Coinfections included hepatitis C virus (genotype 3; viral load: 6.29 × 10⁶ IU/mL) and hepatitis B virus, both confirmed by PCR. Oral candidiasis was diagnosed clinically based on characteristic white plaques on the tongue and oral mucosa, and was further supported by the detection of *Candida *spp*.* at approximately 10⁶ CFU/mL in an oral swab analysis. Liver elastography demonstrated stage III-IV fibrosis. Laboratory findings on admission are summarized in Table 1.

**Table 1:** Laboratory investigations on admission.

Test	Result	Reference range	Interpretation
Complete blood count			
Hemoglobin	109 g/L	130–170 g/L (M)	Mild anemia
White blood cells	3.38 × 10⁹/μL	4.0–10.0 × 10⁹/μL	Leukopenia
Red blood cells	3.86 × 10¹²/L	4.5–5.9 × 10¹²/L	Slightly decreased
Platelets	202 × 10⁹/μL	150–400 × 10⁹/μL	Normal
Neutrophils	3.92 × 10⁹/μL	2.0–7.0 × 10⁹/μL	Normal
Lymphocytes	1.01 × 10⁹/μL	1.0–3.0 × 10⁹/μL	Low-normal
Erythrocyte sedimentation rate	34 mm/hour	<20 mm/hour	Elevated (inflammation)
C-reactive protein	145 mg/L	<5 mg/L	Markedly elevated (acute-phase reactant)
Urinalysis			
Specific gravity	1.028	1.005–1.030	Normal
pH	6.0	4.5–8.0	Normal
Protein	Trace	Negative	Minor proteinuria
Leukocytes	8–12/HPF	0–5/HPF	Pyuria
Erythrocytes	2–4/HPF	0–2/HPF	Microhematuria
Blood biochemistry			
Total bilirubin	12.7 μmol/L	5–21 μmol/L	Normal
Glucose	4.9 mmol/L	3.9–5.8 mmol/L	Normal
Urea	7.4 mmol/L	2.5–7.5 mmol/L	High normal
Creatinine	100 μmol/L	62–106 μmol/L	Normal
Aspartate transaminase	35 U/L	<40 U/L	Normal
Alanine transaminase	56.9 U/L	<41 U/L	Mildly elevated (hepatocellular injury)
Total protein	82.6 g/L	64–83 g/L	Upper normal
Albumin	21.7 g/L	35–52 g/L	Hypoalbuminemia (malnutrition, liver disease)

**Figure 2:**
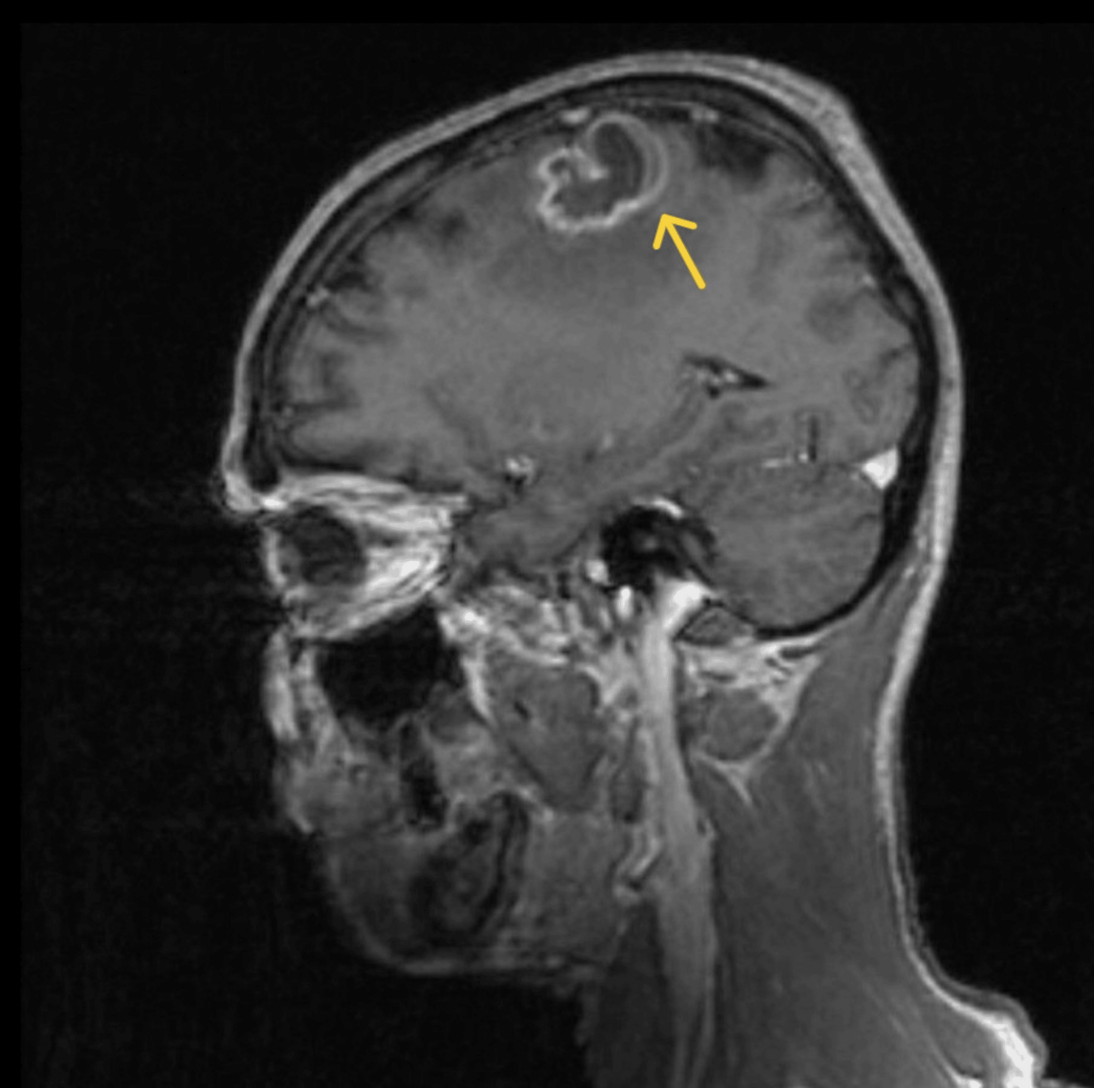
Contrast-enhanced sagittal MRI showing a ring-enhancing lesion with surrounding edema in the left frontal lobe, consistent with a tuberculoma, as indicated by the arrow.

MRI of the brain with contrast identified a lesion in the left frontal lobe with surrounding edema, suggestive of a tuberculoma with signs of liquefaction (Figure 2). An additional minor enhancement was noted in the left occipital lobe, along with several small vascular lesions. The diagnosis was further supported by cerebrospinal fluid analysis, which confirmed the presence of MTB. Neurological consultation confirmed the diagnosis of cerebral tuberculoma, secondary epilepsy, and right-sided hemiparesis.

Two days after admission, the patient developed a papulovesicular rash. Given his immunocompromised status, a clinical diagnosis of varicella was made, and the patient was placed in isolation.

A multidisciplinary team, including specialists in infectious diseases, neurology, radiology, hepatology, and critical care, ultimately confirmed the diagnoses of disseminated TB (pulmonary and cerebral), AIDS, chronic hepatitis B and C with cirrhosis, COVID-19, varicella, oral candidiasis, secondary epilepsy, and pre-XDR tuberculosis.

Anti-tuberculosis treatment was initiated on the third day of admission with a five-drug regimen consisting of bedaquiline, clofazimine, amikacin, delamanid, and linezolid. The patient was planned to receive this regimen for an initial six months, after which the treatment plan would be reassessed. Amikacin was discontinued after two months due to hearing loss, confirmed by audiometry. ART was started two months later, comprising tenofovir, emtricitabine, and dolutegravir.

Following ART initiation, the patient developed IRIS, presenting with fever, abdominal bloating, increased seizure frequency, hypotonia, and cognitive slowing. ART was continued, and corticosteroid therapy was initiated to control the IRIS-related inflammatory response. Corticosteroids were administered for 11 days, followed by a period of dose tapering based on clinical improvement. Abdominal ultrasound revealed hepatomegaly, ascites, and perivascular fibrosis, consistent with hepatic decompensation.

Supportive care included anticonvulsants, antipyretics, nonsteroidal anti-inflammatory drugs, and diuretics. The patient gradually improved, with resolution of seizures, improved neurological function, and reduced hepatic congestion.

A follow-up brain MRI performed five months after admission demonstrated complete radiological resolution of the tuberculoma and the previously noted enhancement in the left occipital lobe. By five and a half months, the patient was discharged on ambulatory treatment with four anti-tuberculosis agents (bedaquiline, clofazimine, delamanid, and linezolid) and continued ART.

Hepatitis C infection was treated with a 24-week course of sofosbuvir and daclatasvir, resulting in virological cure. Final sputum cultures were negative at the time of discharge. Follow-up chest imaging demonstrated resolution of pulmonary infiltrates and cavitary lesions. The patient showed adequate functional and neurocognitive recovery to return to work and remained engaged in outpatient HIV care.

## Discussion

This case illustrates the complexity of managing cerebral tuberculoma in a patient with pre-XDR TB and advanced HIV/AIDS, compounded by multiple viral coinfections. CNS tuberculosis poses both diagnostic and therapeutic challenges due to its paucibacillary nature and diverse clinical manifestations [10]. Diagnosis relies heavily on MRI and microbiological testing from all involved sites [11].

The management of pre-XDR TB involves the use of potent second-line agents such as bedaquiline, linezolid, and delamanid, as demonstrated in this case. However, these regimens carry a significant risk of adverse effects. Bedaquiline has been associated with acute liver injury, while delamanid increases the risk of long QT-related cardiac events, particularly within the first six months of therapy [12]. These risks necessitate close clinical and laboratory monitoring, especially in patients with underlying liver dysfunction or severe immunosuppression. Notably, amikacin was discontinued due to ototoxicity [13], underscoring the need for vigilant adverse effect surveillance.

In this case, ART was initiated approximately two months after the start of anti-tuberculosis treatment, following the Adult and Adolescent Antiretroviral Guidelines, which recommend initiating ART within two to eight weeks of starting TB treatment in patients with HIV-TB coinfection and CD4 counts ≥50 cells/mm³ [14]. Despite this timing, the patient developed IRIS, highlighting that it can still occur in severely immunocompromised individuals, particularly those with CNS involvement. The decision regarding ART timing in such cases must balance the risk of delaying ART with the risk of unmasking or worsening opportunistic infections. According to current expert guidance, ART should ideally be initiated after stabilization of opportunistic infections and before profound immunosuppression sets in. A thorough evaluation for latent or active infections before ART initiation is essential, especially in patients at high risk for IRIS [15].

The presence of chronic hepatitis B and C, COVID-19, and reactivated varicella added further complexity, increasing the risk of hepatotoxicity and drug-drug interactions. Managing such coinfections requires a multidisciplinary approach and individualized treatment planning to optimize outcomes.

Ultimately, this case underscores the importance of early diagnosis, appropriate antimicrobial selection, and coordinated care in managing complex patients with drug-resistant TB and advanced immunosuppression. Heightened clinical suspicion and the use of advanced imaging modalities are vital in improving the prognosis of CNS tuberculosis.

## Conclusions

This case highlights the complex interplay of cerebral tuberculoma, pre-XDR TB, and advanced HIV/AIDS complicated by multiple viral coinfections. Early neuroimaging is essential in high-risk immunocompromised patients presenting with neurological symptoms. Careful timing of ART, with consideration of IRIS risk, alongside individualized second-line anti-tuberculosis regimens, was pivotal to the favorable outcome observed over six months. Successful management, defined by resolution of neurological symptoms, radiologic improvement, and return to independent functioning, requires multidisciplinary coordination and close monitoring to navigate the challenges of coexisting infections and drug toxicities. This case supports the need for broader screening and earlier intervention among immunocompromised individuals to facilitate timely diagnosis and treatment. Clinicians should maintain a high index of suspicion for CNS tuberculosis in immunosuppressed patients who have neurological symptoms to improve outcomes in this vulnerable population.
